# Otx2 Is Involved in the Regional Specification of the Developing Retinal Pigment Epithelium by Preventing the Expression of Sox2 and *Fgf8*, Factors That Induce Neural Retina Differentiation

**DOI:** 10.1371/journal.pone.0048879

**Published:** 2012-11-08

**Authors:** Daisuke Nishihara, Ichiro Yajima, Hiromasa Tabata, Masato Nakai, Nagaharu Tsukiji, Tatsuya Katahira, Kazuhisa Takeda, Shigeki Shibahara, Harukazu Nakamura, Hiroaki Yamamoto

**Affiliations:** 1 Department of Developmental Biology and Neurosciences, Graduate School of Life Sciences, Tohoku University, Sendai, Miyagi, Japan; 2 Unit of Environmental Health Sciences, Department of Biomedical Sciences, College of Life and Health Sciences, Chubu University, Kasugai, Aichi, Japan; 3 Faculty of Bioscience, Nagahama Institute of Bio-Science and Technology, Nagahama, Shiga, Japan; 4 Laboratory of Developmental Neurobiology, Graduate School of Brain Science, Doshisha University, Kyotanabe, Kyoto, Japan; 5 Department of Molecular Biology and Applied Physiology, Tohoku University School of Medicine, Sendai, Miyagi, Japan; 6 Department of Molecular Neurobiology, Graduate School of Life Sciences and Institute of Development, Aging and Cancer, Tohoku University, Sendai, Miyagi, Japan; Indiana University, United States of America

## Abstract

The retinal pigment epithelium (RPE) shares its developmental origin with the neural retina (NR). When RPE development is disrupted, cells in the presumptive RPE region abnormally differentiate into NR-like cells. Therefore, the prevention of NR differentiation in the presumptive RPE area seems to be essential for regionalizing the RPE during eye development. However, its molecular mechanisms are not fully understood. In this study, we conducted a functional inhibition of a transcription factor Otx2, which is required for RPE development, using early chick embryos. The functional inhibition of *Otx2* in chick eyes, using a recombinant gene encoding a dominant negative form of Otx2, caused the outer layer of the optic cup (the region forming the RPE, when embryos normally develop) to abnormally form an ectopic NR. In that ectopic NR, the characteristics of the RPE did not appear and NR markers were ectopically expressed. Intriguingly, the repression of *Otx2* function also caused the ectopic expression of *Fgf8* and Sox2 in the outer layer of the optic cup (the presumptive RPE region of normally developing eyes). These two factors are known to be capable of inducing NR cell differentiation in the presumptive RPE region, and are not expressed in the normally developing RPE region. Here, we suggest that *Otx2* prevents the presumptive RPE region from forming the NR by repressing the expression of both *Fgf8* and Sox2 which induce the NR cell fate.

## Introduction

The retinal pigment epithelium (RPE), one component of the vertebrate eye, consists of a monolayer of melanin-producing cells. Both the RPE and the neural retina (NR), which contains photoreceptors, retinal ganglion cells (RGC), horizontal cells, amacrine cells, bipolar cells and Müller glia cells, originate from the same eye primordium, called the optic vesicle (OV), which derives from the lateral wall of the forebrain. The inductive interactions between the OV and the surface ectoderm (the future lens) result in the invagination of the OV to form the bilayered optic cup (OC), in which the outer and inner layers are specified into the RPE and NR, respectively [1], [2].

The development of the RPE is promoted by several transcription factors, which are specifically expressed in the presumptive RPE region; *Microphthalmia-associated transcription factor* (*Mitf*) and *Orthodenticle homeobox 1* and *2* (*Otx1* and *2*). Mitf promotes melanin synthesis and regulates cell proliferation in the developing RPE [3]. In mutant mice with non-functional alleles of the *Mitf* gene, a non-pigmented NR-like tissue is ectopically formed in the outer layer of the OC [4], [5]. The expression of *Mitf* in the presumptive RPE region requires the function of *Otx* genes [6]. Compound mutations in *Otx1* and *2* (all *Otx1^−/−^*; *Otx2^+/−^* mice and 30% of *Otx1^+/−^*; *Otx2^+/−^* mice) result in the down-regulation of *Mitf* expression and the ectopic formation of NR-like tissue in the outer layer of the OC, although *Otx1^−/−^* mice do not display significant defects in the RPE [6]. Still, in spite of these key findings in mutant mice, it is unclear whether the loss-of-function of *Otx2* affects RPE development, since the head region including the eyes is not formed in *Otx2^−/−^* mice [7], [8]. However, previous reports have pointed out the roles of Otx2 as an upstream regulator of *Mitf* expression and the promotion of RPE differentiation [9], [10]. In cultured quail retina cells, transfection of *Otx2* induces a pigmented phenotype with Mitf expression [9]. In the chick NR, co-transfection of *Otx2* and a constitutively active form of *β-catenin* induces the ectopic expression of Mitf [10].

While RPE development requires the functions of *Mitf* and *Otx*, NR differentiation is induced by several other transcription and growth factors, including Fibroblast growth factor 8 (Fgf8), Subgroup B1 SRY-box family genes (SoxB1) and Paired-box 6 (Pax6). When FGF8-soaked beads are placed in the vicinity of the developing RPE, cells in the RPE change their fate to differentiate into NR cells [11]. As a result, some areas of the outer layer of the OC form a non-pigmented ‘ectopic NR’ which takes on a stratified structure and displays several differentiation markers of the NR [11]. Similarly, ectopic formation of the NR can also be caused by the misexpression of *SoxB1* or *Pax6* in the outer layer of the OC [12], [13]. The *Fgf8* and *SoxB1* genes are expressed in the NR, but not in the RPE [11], [12]. Pax6 also becomes absent from the presumptive RPE, although its expression is detected in the RPE during the early stages of eye development [14], [15]. Although the expression patterns of these factors are well known, it is noteworthy that it is still unclear how these factors are restricted to the NR region and disappear from the RPE region in normally developing eyes. Unveiling how the expression domains of *Fgf8*, SoxB1 and Pax6 are down-regulated in the outer layer of the OC may lead to understanding the mechanism(s) involved in the regionalization of the RPE.

Since Otx2 and Mitf are specifically expressed in the outer layer of the OC (the region forming the RPE, when embryos normally develop), it is possible that these RPE-specific factors are involved in the down-regulation of *Fgf8*, SoxB1 and Pax6 in the outer layer of the OC. Here, we conducted a functional inhibition of Otx2 in the developing eye, using chick embryos. Chick embryos were transfected with a recombinant gene encoding a dominant negative form of Otx2 that was confirmed to repress the function of wild-type *Otx2 in vitro*. Functional inhibition of Otx2 in chick eyes caused the outer layer of the optic cup (the RPE region of normally developing eyes) to abnormally form an ectopic NR. In that ectopic NR, the characteristics of the RPE did not appear and NR markers were ectopically expressed. Intriguingly, the repression of Otx2 function also caused the ectopic expression of *Fgf8* and Sox2 (one of the SoxB1 family members) in the outer layer of the OC, whereas the expression of Pax6 was reduced. Our data suggest that *Otx2* prevents the outer layer of the OC from forming the NR by repressing the expression of *Fgf8* and Sox2 which can forcedly induce NR differentiation [11], [12].

## Results

### Expression Pattern of *Otx2* and the Dominant Negative Activity of *EnR-Otx2*

**Figure 1:**
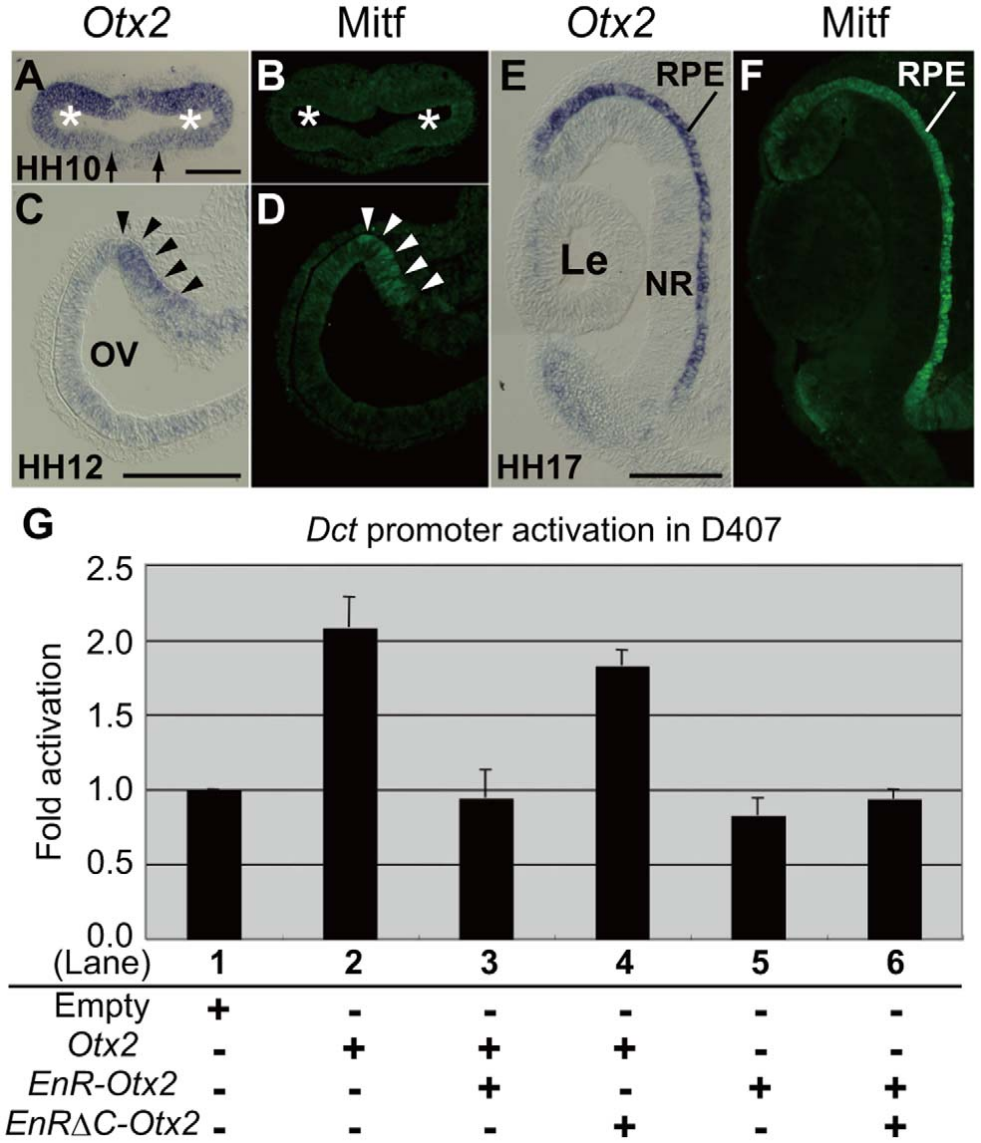
Expression pattern of *Otx2* and the dominant negative activity of *EnR-Otx2.* (A–F) *In situ* hybridization analyses of *Otx2* expression and immunohistological analyses of Mitf expression. A and B are serial sections of an HH10 embryo, as well as C and D of an HH12 embryo, and E and F of an HH17 embryo. A, C and E show expression of *Otx2*, and B, D and F show Mitf staining. Asterisks in A and B indicate the OV. Arrows in A indicate the ventral area of the OV where *Otx2* is weakly expressed. Arrowheads in C and D highlight the sites where *Otx2* and Mitf are strongly expressed. Upper and lower sides of panels A–F correspond to dorsal and ventral sides of the embryos, respectively. (G) *Dct* promoter activity in D407 cells. The *Dct* promoter fused to *luciferase* was co-transfected with the vectors, as follows. Co-transfection with: empty vector (Lane 1), *Otx2* (Lane 2), *Otx2* and *EnR-Otx2* (Lane 3), *Otx*2 and *EnRΔC-Otx2* (Lane 4), *EnR-Otx2* (Lane 5) and *EnR-Otx2* and *EnRΔC-Otx2* (Lane 6). The histogram presents means ± SD. RPE, retinal pigment epithelium. NR, neural retina. Le, lens. Scale bars: 100 µm.

First, we compared the expression patterns of *Otx2* with Mitf in the OV and the OC stage. In HH10 chick embryos, *Otx2* was expressed in a large part of the OV (asterisks in Figure 1A), although its expression was weak in the ventral part of the OV. Mitf was not expressed in the OV in HH10 chick embryos (Figure 1B). From HH12-13, Mitf expression could be detected in the dorsal part of the OV (arrowheads in Figure 1D). At the same stages, *Otx2* was highly expressed in the dorsal part of the OV (arrowheads in Figure 1C), similar to the expression pattern of Mitf. After the OC was formed, the expression of both *Otx2* and Mitf was apparent in the outer layer of the OC where the RPE formed (Figure 1E and F).

For the functional inhibition of *Otx2*, we used a recombinant gene encoding a dominant negative form of Otx2, called *EnR-Otx2*. *EnR-Otx2* encodes chick Otx2 fused to the *Drosophila* Engrailed repressor domain (EnR). We confirmed the dominant negative activity of *EnR-Otx2* with *in vitro* assays. Consistent with a previous study which showed that OTX2 activates the *Dct* gene promoter [16], chick wild-type *Otx2* (wt*Otx2*) drove the *Dct* promoter (lanes 1 and 2 in Figure 1G) in D407 cultured cells. This function of *wtOtx2* was blocked by *EnR-Otx2* (lane 3 in Figure 1G), suggesting that *EnR-Otx2* could be used for the functional inhibition of *Otx2*. As expected, the *Dct* promoter was not activated by *EnR-Otx2* (lane 5 in Figure 1G). *EnR-Otx2ΔC*, which encodes EnR-Otx2 without its C-terminal DNA-binding domain, did not repress the activity of *wtOtx2* (lane 4 in Figure 1G).

### Loss of Characteristics of the RPE by *EnR-Otx2* Transfection

To address how *Otx2* contributes to RPE development in chick embryos, we conducted gene transfection experiments. The OV of HH9-11 chick embryos (incubated for 1.5 days) were transfected with pMiwIII-*EnR-Otx2* and a GFP-expressing vector (*pCAGGS-EGFP*) using *in ovo* electroporation, carried out as described previously [3]. For controls, both the pMiwIII-empty vector and *pCAGGS-EGFP* were transfected. After incubation for 2 days, the transfected embryos were fixed and prepared for observation.

**Figure 2:**
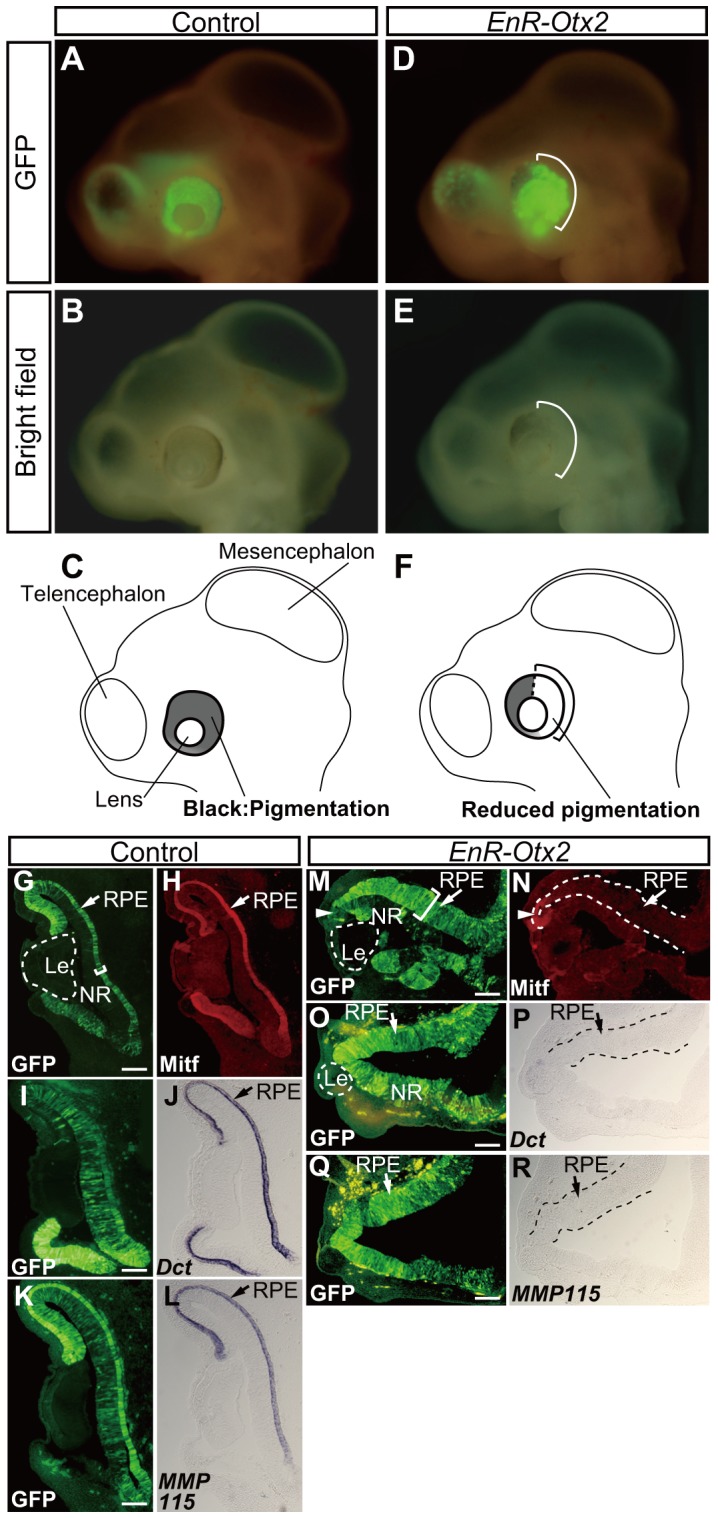
Loss of RPE characteristics and morphological changes in the chick eye following *EnR-Otx2* transfection. (A–F) Lateral views of embryos transfected with either the empty vector (A and B), or *EnR-Otx2* (D and E). Illustrations in C and F correspond to B and E, respectively. Brackets in D and E indicate sites where pigmentation was reduced by *EnR-Otx2*. A and D indicate GFP signals (green). B and E are bright field images. (G–R) Staining of RPE markers in sections of eyes transfected with the empty vector (G–L) or *EnR -Otx2* (M–R). Immunohistological analyses of Mitf expression (H and N) and *in situ* hybridization analyses of transcripts of *Dct* (J and P) or *MMP115* (L and R). G, I, K, M, O and Q indicate GFP signals (green). Brackets in G and M indicate the thickness of the outer layer of the OC, which are transfected with the empty vector (G) or *EnR-Otx2* (M), respectively. Dashed lines in N, P and R highlight the thickened outer layer of *EnR-Otx2*-transfected eyes. Arrowheads in M and N indicate sites where Mitf expression remains. In M–R, ‘RPE’ refers to the abnormally thickened outer layer caused by *EnR-Otx2* transfection. G and H are the same sections, so are M and N. Each set of I and J, K and L, O and P and Q and R are serial sections. Upper and lower sides of the panels correspond to dorsal and ventral sides of embryos, respectively. RPE, retinal pigment epithelium. NR, neural retina. Le, lens. Scale bars: 100 µm.

As in the case of normally developing eyes, the control eyes had a blackish tinge except for the lens (n = 15), since the differentiated RPE cells synthesize melanin pigment (corresponding to HH20-22 embryos, Figure 2A–C). However, the *EnR-Otx2*-transfected eyes revealed a highly reduced pigmentation in the GFP-positive portion (n = 23, brackets in Figure 2D–F).

To address how *EnR-Otx2* affects the state of differentiation and morphology of the outer layer of the OC (the presumptive RPE region of normally developing eyes), sections of *EnR-Otx2*-transfected or control eyes were stained for RPE differentiation markers. In the control eyes, no obvious morphological changes were observed (Figure 2G–L), and the outer layer of the OC (the presumptive RPE region) maintained the mono-layered structure (bracket in Figure 2G). In contrast, *EnR-Otx2* caused an abnormally thickened tissue to be formed in the outer layer of the OC (compare brackets between Figure 2G and M). In this thickened outer layer (the areas sandwiched between the dashed lines), pigment granules were hardly detected. In addition, some of the *EnR-Otx2* transfected eyes did not keep their cup-like structure to form an OV-like structure (Figure 2O–R), and the size of the lens seemed reduced in the *EnR-Otx2-*transfected eyes (compare the areas enclosed in the dashed lines in Figure 2M and O with Figure 2G).

In *EnR-Otx2*-transfected eyes (Figure 2M–R), the expression of Mitf became weakened over a large part of the thickened outer layer (Figure 2M and N), and Mitf signals were only detected in the GFP-negative areas in the thickened outer layer (arrowheads in Figure 2M and N). Differentiation markers of the RPE, *dopachrome tautomerase* (*Dct,* encoding a enzyme required for black melanin synthesis) and *melanosomal matrix protein 115* (*MMP115*) [3], [17], [18], also could not be detected in these eyes (the areas between the dashed lines in Figure 2P and R). In contrast, Mitf, *Dct* and *MMP115* were specifically expressed in the outer layer of the OC (the presumptive RPE region) of control eyes (Figure 2G–L). Thus, proper morphogenesis and differentiation in the outer layer of the OC (the presumptive RPE region) is disrupted by *EnR-Otx2* misexpression.

### Ectopic Expression of NR Markers in the Outer Layer of the OC Following *EnR-Otx2* Transfection

Next, we examined the expression patterns of several NR markers. It is possible that *EnR-Otx2* transfection caused the outer layer of the OC to abnormally differentiate into the NR instead of the RPE, since ectopic NR-like tissues are formed in the outer layer of the OC of *Otx1* and *Otx2* compound mutant mice [6]. In addition, previous studies have shown that the RPE shares a common developmental origin (OV) with the NR and also has the potential to differentiate into the NR [11], [12], [13], [19], [20], [21], [22], [23], [24], [25], [26].

For analyzing the expression of NR markers, the embryos were transfected at embryonic stages HH9-11, and then were further incubated for 2 days to reach HH20-22 embryos.

**Figure 3:**
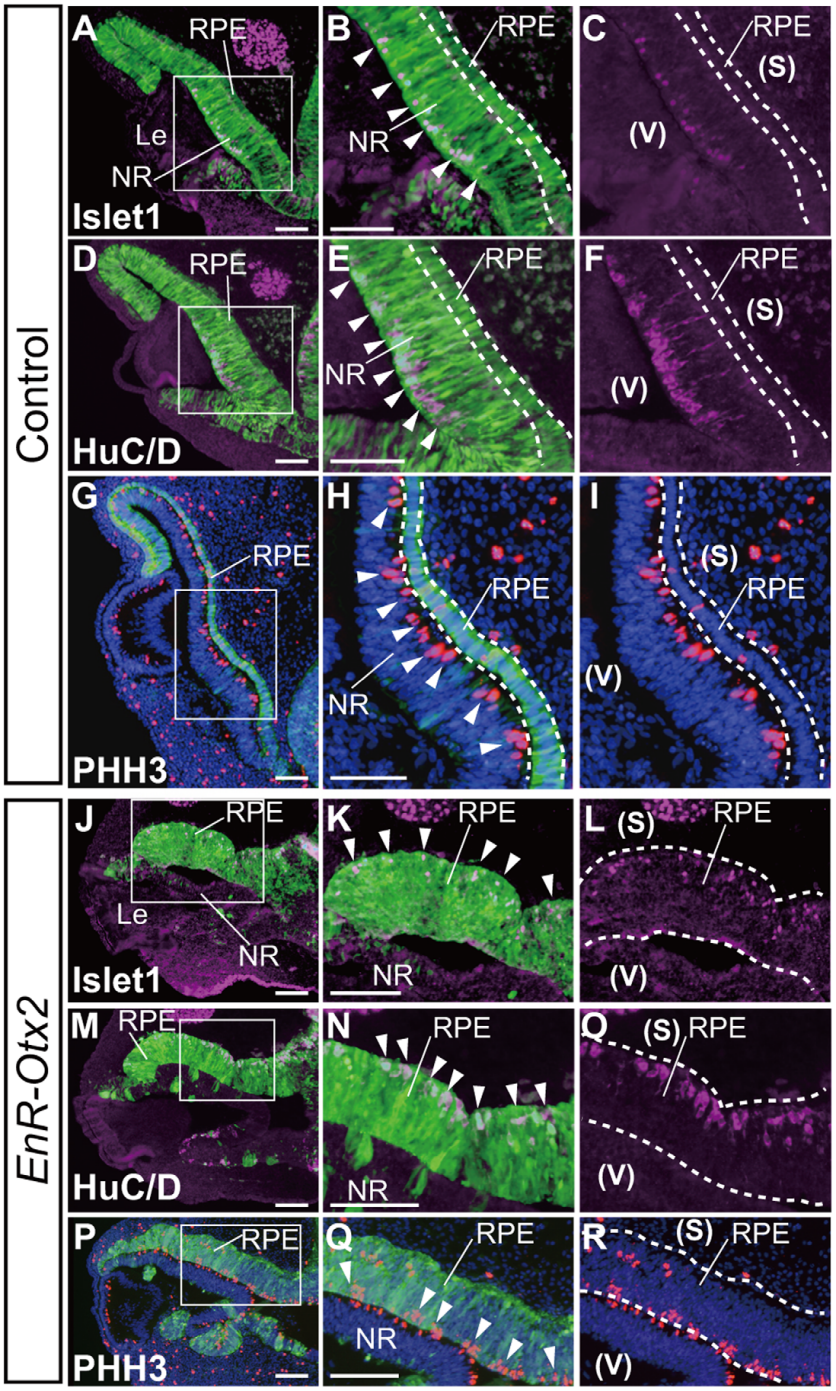
Ectopic expression of NR markers in the outer layer of the OC following *EnR-Otx2* transfection. Immunohistological staining of NR markers in sections of eyes transfected with the empty vector (A–I) or *EnR -Otx2* (J–R). A–C and J–L indicate the expression of Islet1 (magenta), and A, B, J and K are merged images with GFP (green). D–F and M–O indicate the expression of HuC/D (magenta), and D, E, M and N are merged images with GFP (green). G–I and P–R indicate signals of phospho-Histone H3 (PHH3, red) and nuclei (DAPI, blue) and G, H, P and Q are merged images with GFP (green). B and C, E and F, H and I, K and L, N and O, and Q and R are magnified images of the boxes in A, D, G, J, M and P, respectively. Dashed lines highlight the RPE of control eyes (B, C, E, F, H and I) or the thickened outer layer of *EnR-Otx2*-transfected eyes (K, L, N, O, Q and R). Arrowheads indicate the positions of cells which are positive for Islet1 (B and K), HuC/D (E and N) or PHH3 (H and Q). (v) and (s) in C, F, I, L, Q and R indicate the vitreal and sclera sides of the OC, respectively, across the inner and outer layers of the OC. DAPI is used to ease observation of tissues (G–I and P–R). The upper and lower sides of each image correspond to the dorsal and ventral sides of the specimen, respectively. In J-R, ‘RPE’ refers to the abnormally thickened outer layer, apparently ‘ectopic NR’. RPE, retinal pigment epithelium. NR, neural retina. Le, lens. Scale bars: 100 µm.

In the developing NR, the transcription factor Islet1 is detected in postmitotic ganglion cells, migrating amacrine cells [11], [12], [27], while RNA-binding protein HuC/D is expressed in the differentiated neuronal cells [21], [28], [29]. In the control eyes, those differentiation markers of the NR were expressed on the vitreal surface (the surface facing the lens, described as (v) in Figure 3C and F) of the NR (arrowheads in Figure 3B and E), but could not be detected in the outer layer of the OC/presumptive RPE region (the area between the dashed lines in Figure 3B, C, E and F). In addition, phospho histone-H3 (PHH3)-positive mitotic cells were located at the sclera surface (the surface opposite the lens, described as (s) in Figure 3I) of the NR in the control eyes (arrowheads in Figure 3H), as in the case of normally developing eyes. In these eyes, only a small number of RPE cells were positive for PHH3 (the area between the dashed lines in Figure 3H and I).

In the *EnR-Otx2*-transfected eyes, not only the NR but also the thickened outer layer were positive for HuC/D and Islet1 (Figure 3J–Q). Ectopic signals of Islet1 or HuC/D were detected on the sclera surface (the surface opposite the lens, described as (s) in Figure 3L and Q) of the thickened outer layer (arrowheads in Figure 3K and N). Further, on the vitreal surface (the surface facing the lens, described as (v) in Figure 3R) of the thickened outer layer, most PHH3-positive cells were detected (arrowheads in Figure 3Q). The distribution of PHH3 on the opposite side of HuC/D and Islet1 in the thickened outer layer topologically mimicked the distribution of those factors in the normally developing NR. These data suggest that *EnR-Otx2* misexpression results in the formation of an ‘ectopic NR’ in the outer layer of the OC.

### Effects of *EnR-Otx2* on Factors Involved in NR Differentiation or other Aspects of Eye Development

The ectopic NR formation in the outer layer of the OC (the presumptive RPE region) could also be induced by some transcription factors or secreted factors, which are expressed in the OC. For example, misexpression of transcription factors *Sox1*, *2* or *3* (SoxB1) or *Pax6* causes ectopic formation of the NR in the outer layer of the OC (the presumptive RPE region) [12], [13]. The application of FGF8-soaked beads in the vicinity of the developing RPE also results in the ectopic NR formation [11].

In normally developing eyes, the expression of *Sox1*, *2* and *3* (SoxB1), *Pax6* and *Fgf8* is detected in the NR [11], [12], [14], [15]. It is noteworthy that *SoxB1* and *Fgf8* are not expressed in the normally developing RPE after the OC is formed [11], [12]. Although Pax6 is expressed in the normally developing RPE after the OC formation, its expression disappears from the presumptive RPE region as eye development further proceeds [14], [15].

In other words, the expression domains of *SoxB1*, *Pax6* and *Fgf8*, which induce NR differentiation eventually become restricted to the presumptive NR region during normal eye development. In this point of view, we tested whether expression of these factors was induced in the ectopic NR by *EnR-Otx2* transfection.

For this analysis, the embryos were transfected in embryonic stages HH9-11, and then were further incubated for 2 days to reach HH20-22 embryos.

**Figure 4:**
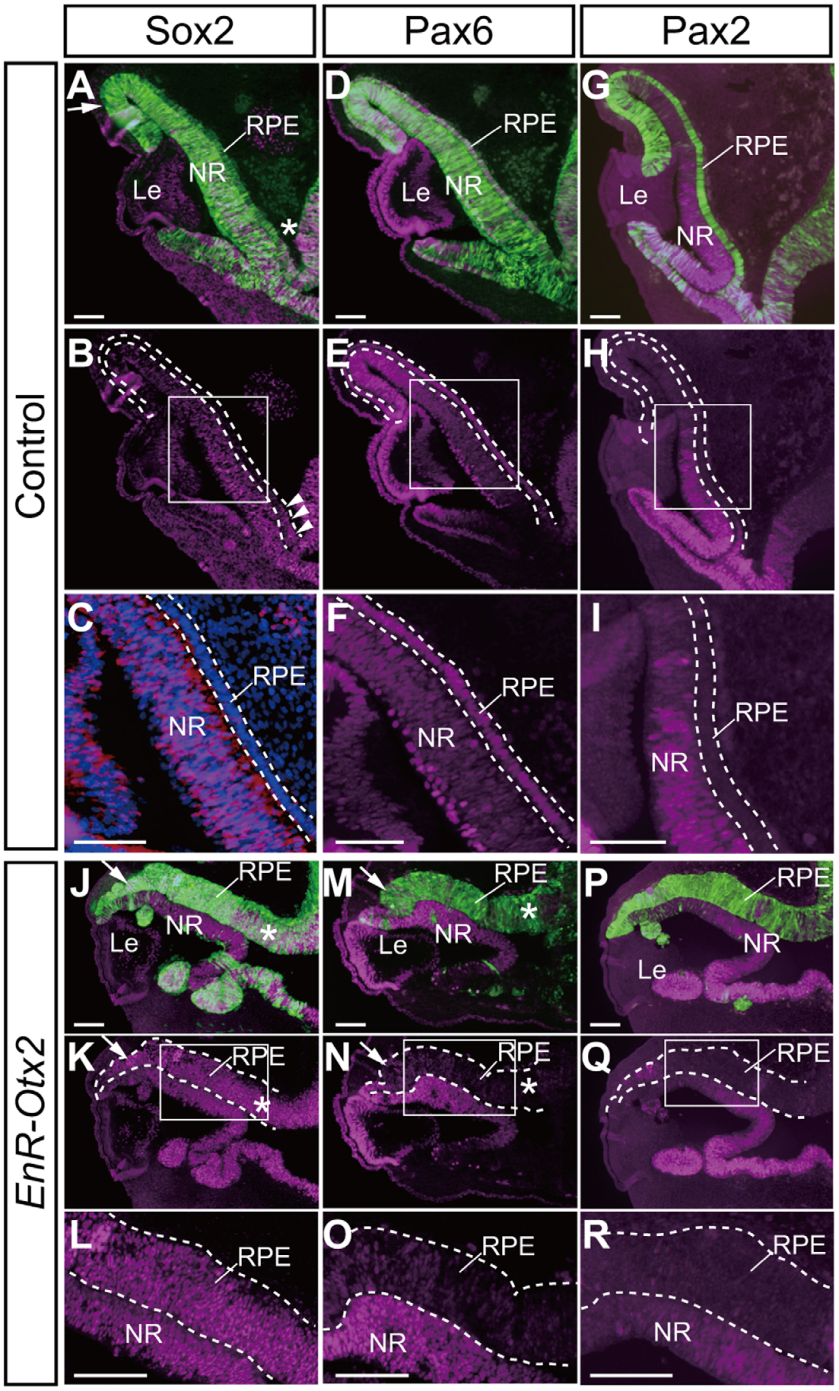
Alterations of expression patterns of transcription factors following *EnR-Otx2* transfection. Immunohistological staining of Sox2, Pax6 and Pax2 in sections of eyes transfected with empty vector (A–I) or *EnR -Otx2* (J–R). A–C and J–L indicate the expression of Sox2 (magenta). A and J are merged images with GFP (green). DAPI (blue) in C is used to ease observation of tissue structures of the RPE and NR. D–F and M–O indicate the expression of Pax6 (magenta). D and M are merged images with GFP (green). G–I and P–R indicate the expression of Pax2 (magenta). G and P are merged images with GFP (green). C, F, I, L, O and R are magnified images of the boxes in B, E, H, K, N and Q, respectively. Dashed lines highlight the RPE of control eyes (B–I) or the thickened outer layer of *EnR -Otx2*-transfected eyes (K–R). Arrows and asterisks in A, J, K, M and N indicate the peripheral and proximal areas of the outer layer of the OC, respectively. The central area of the outer layer of the OC corresponds to the area between the arrow and the asterisk. Arrowheads in B highlight the Sox2-positive small area of the RPE. The upper and lower sides of each image correspond to the dorsal and ventral sides of the specimen, respectively. In J–R, ‘RPE’ refers to the abnormally thickened outer layer, apparently ‘ectopic NR’. RPE, retinal pigment epithelium. NR, neural retina. Le, lens. Scale bars: 100 µm.

In the control eyes, Sox2 was not expressed in a large part of the outer layer of the OC (the RPE region, the areas between the dashed lines in Figure 4B and C), but its expression was detected in the NR (Figure 4A–C). In these eyes, Sox2 was not expressed in the peripheral (arrow in Figure 4A) or central RPE, although only a small part of the proximal RPE (asterisk in Figure 4A) was positive for Sox2 signals (arrowheads in Figure 4B). In contrast, transfection of *EnR-Otx2* caused the ectopic expression of Sox2 in the thickened outer layer of the OC/ectopic NR (the areas between the dashed lines in Figure 4K and L). The expression of Sox2 was detected in the peripheral, central and proximal regions of the thickened outer layer/ectopic NR following *EnR-Otx2* transfection (Figure 4J–L). Moreover, unlike in the cases of Islet1 and HuC/D, Sox2 was not only expressed in the basal side but over the whole range of the ectopic NR across the apical-basal axis (Figure 4K and L).

**Figure 5:**
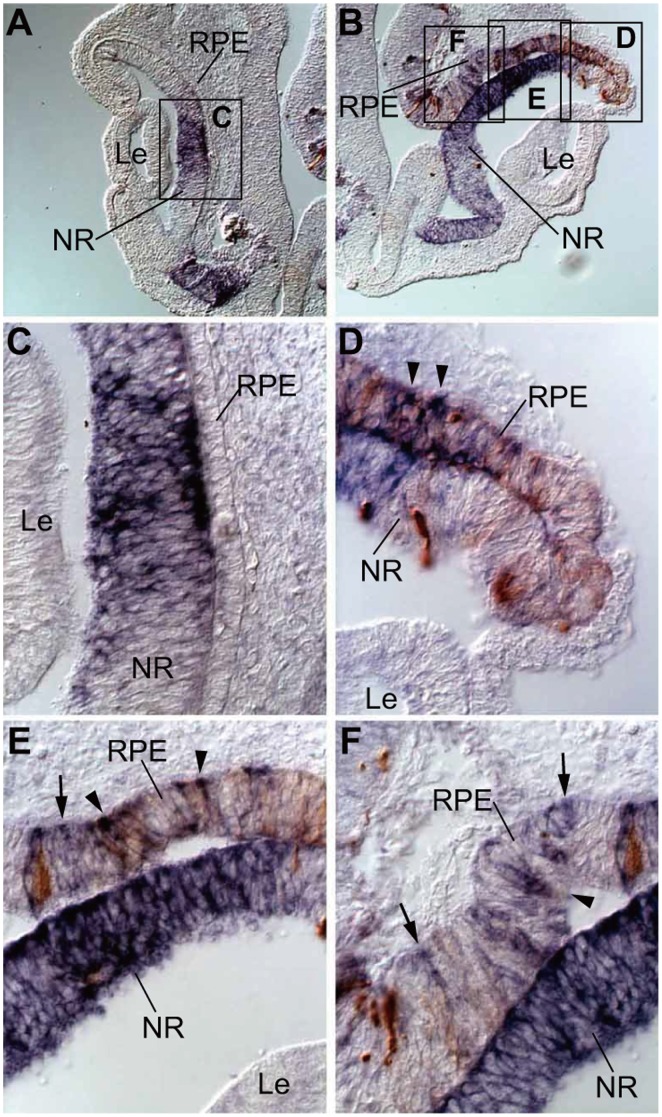
Alteration of expression pattern of *Fgf8* following *EnR-Otx2* transfection. In situ hybridization analyses of the expression of *Fgf8* in sections of normally developing eyes (A and C) and *EnR-Otx2*-transfected eyes (B and D–F). Expression of GFP is indicated as a brown signal in B and D–F. C is a highly magnified image of the box in A. D–F are highly magnified images of the boxes in B. Arrowheads in D–F indicate sites where *Fgf8* and GFP signals overlap. Arrows in E and F indicate *Fgf8*-positive areas in which GFP signals are relatively weak. The upper and lower sides of each image correspond to the dorsal and ventral sides of the specimen, respectively. In B and D–F, ‘RPE’ refers to the abnormally thickened outer layer, apparently ‘ectopic NR’. RPE, retinal pigment epithelium. NR, neural retina. Le, lens.

Similar to Sox2, *EnR-Otx2* induced the ectopic expression of *Fgf8* in the thickened outer layer of the OC/ectopic NR (Figure 5B and D–F). In the normal eyes, *Fgf8* was expressed in the central part of the NR but not in the outer layer of the OC/presumptive RPE region (Figure 5A and C). Interestingly, ectopic expression of *Fgf8* was also detected in the dorsal and ventral parts of the NR by *EnR-Otx2*, although only a few NR cells were transfected with *EnR-Otx2* (Figure 5B).

In contrast to Sox2 and *Fgf8*, Pax6 was strongly expressed in the outer layer of the OC (the presumptive RPE region) of control embryos, which were incubated for 2 days after electroporation (corresponding to HH20-22 embryos, Figure 4D–F). In the normally developing HH20-22 embryos, Pax6 was similarly detected in the outer layer of the OC (the presumptive RPE region). It was intriguing that *EnR-Otx2* transfection resulted in the reduced expression of Pax6 in the thickened outer layer of the OC/ectopic NR (Figure 4M–O). Pax6 expression was apparently weakened in the proximal part of the thickened outer layer/ectopic NR (asterisks in Figure 4M and N), and expression of Pax6 also became weaker in the peripheral part of the thickened outer layer/ectopic NR (arrows in Figure 4M and N).

We also analyzed whether some other transcription factors changed their expression patterns in the outer layer of the OC following *EnR-Otx2* transfection. We examined the expression of *Six3*, *Lhx2* and Pax2, each of which is associated with multiple aspects of eye development.

During eye development, inactivation of *Six3* causes cyclopia, small eyes or disrupted proximo-distal patterning of the OV in medaka embryos [30]. Over-expression of *Six3* results in retinal hyperplasia or ectopic retinal primordia formation [31]. In the postnatal retina, *Six3* is also involved in cell specification [32], [33].

In the case of eye development in *Lhx2*, *Lhx2^−/−^* mice have eye development that is arrested in the OV stage, and expression domains of various transcription factors are disrupted until the OV stages [34], [35]. To analyze the function of *Lhx2* in OC stages, Yun et al. generated genetic mosaic mice, in which *Lhx2*-mutant cells exist at low frequency among the wild-type cells [35]. In the OC of these mice, Mitf, Vsx2 and Pax2 were not expressed in the *Lhx2*-mutant cells, although Pax6 was expressed [35].

Pax2 is required for optic fissure closure and proper projection of the optic nerve [36], [37]. Although Pax2 is not expressed in the presumptive RPE, its expression is detected in the optic stalk (OS), which is adjacent to the presumptive RPE [36], [38]. *Pax2*-deficient mutant mice display expansion of the RPE domain toward the OS region [36], [38]. Therefore, Pax2 is thought to repress RPE development to make a sharp boundary between the OS and the RPE [38].

**Figure 6:**
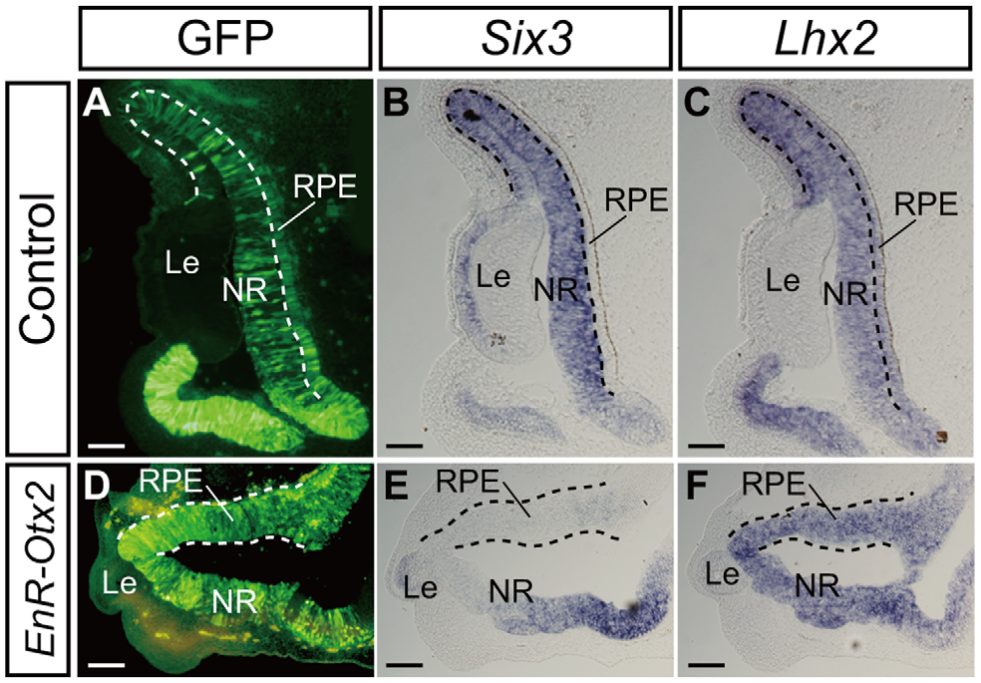
Expression patterns of *Six3* and *Lhx2* in *EnR-Otx2*-transfected eyes. *In situ* hybridization analyses of the expression of *Six3* (B and E) and *Lhx2* (C and F) in sections of eyes transfected with an empty vector (A–C) or *EnR-Otx2* (D–F). A and D indicate GFP signals (green). A–C and D–F are serial sections. Dashed lines in A–C indicate boundaries between the RPE and NR. Dashed lines in D–F highlight the thickened outer layer of *EnR-Otx2*-transfected eyes. The upper and lower sides of each image correspond to the dorsal and ventral sides of the specimen, respectively. In D-F,‘RPE’ refers to the abnormally thickened outer layer, apparently ‘ectopic NR’. RPE, retinal pigment epithelium. NR, neural retina. Le, lens. Scale bars: 100 µm.

In the control eyes, expression of Pax2 and *Six3* was not detected in the outer layer of the OC (the presumptive RPE region) (the areas between the dashed lines in Figure 4H and I and described as ‘RPE’ in Figure 6A and B, respectively), although *Lhx2* was expressed in this region (described as ‘RPE’ in Figure 6C). Similarly, the ectopic NR was negative for Pax2 and *Six3* (the areas between the dashed lines in Figure 4Q and R and Figure 6E, respectively) but was positive for *Lhx2* expression in the *EnR-Otx2* transfected eyes (the area between the dashed lines in Figure 6F). In the control NR, *Six3* and *Lhx2* were expressed (Figure 6A–C), although Pax2 was highly expressed in the ventral NR but was only weakly expressed in the dorsal NR (Figure 4G–I). Similar expression patterns of Pax2, *Six3* and *Lhx2* were observed in the NR of *EnR-Otx2* transfected eyes (Figure 4P–R and Figure 6D–F).

Our analyses of the transcription factors and a secreted factor suggest that *EnR-Otx2* induces the ectopic expression of Sox2 and *Fgf8* in the thickened outer layer of the OC/ectopic NR. It is notable that these two factors share two traits; 1) being able to forcedly induce NR differentiation in the outer layer of the OC/presumptive RPE region [11], [12], and 2) being not expressed in the outer layer of the OC/presumptive RPE region but detected in the inner layer of the OC/presumptive NR region in normally developing eyes [11], [12].

### Increased Cell Proliferation and Apoptosis in *EnR-Otx2*-transfected Eyes

Martinez-Morales et al. reported that *Otx1^−/−^; Otx2^+/−^* mice display increased cell proliferation and cell death in the retina [6]. Therefore, we also assessed the effects of *EnR-Otx2* on cell proliferation and apoptosis in chick eyes. To analyze cell proliferation and apoptosis, anti-PHH3 and anti-single stranded DNA (ssDNA) antibodies were used, respectively.

For this analysis, the embryos were transfected in embryonic stages HH9-11, and then were further incubated for 2 days to reach HH20-22 embryos.

Although a small number of PHH3-positive or ssDNA-positive cells existed in the normal RPE and NR (Figure S1A, D, F and I), the number increased following *EnR-Otx2* transfection (Figure S1B, C, E, G, H and J), as in the case of *Otx* mutant mice [6].

### Ectopic Formation of Telencephalon-like Vesicles Following *EnR-Otx2* Transfection

When embryos were incubated for about a week after *EnR-Otx2* transfection, their eyes displayed a small eye phenotype (Figure S2A), as in the case of *Otx1^−/−^; Otx2^+/−^* mice. Intriguingly, we noticed that some vesicle-like structures were ectopically formed following *EnR-Otx2* transfection (arrow in Figure S2A). The ectopic vesicles were connected to the small eyes of *EnR-Otx2* transfected embryos, but were not observed in control embryos (data not shown). The ectopic vesicles lacked the characteristics of the RPE (pigmentation and a monolayered-structure, Figure S2B, C, F, G, I and J) or the NR (expression of a photoreceptor marker visinin, Figure S2C).

These ectopic vesicles were positive for markers of the developing brain, including *Emx1*, *Nkx2.1* and Pax6 (Figure S2B and F–K), but were negative for a hindbrain marker, *Gbx2* (data not shown). *Emx1* is normally detected in the dorsal telencephalon [39], [40], Nkx2.1 in the hypothalamus in the ventral portion of the diencephalon and telencephalon [41] and Pax6 in the dorsal telencephalon and diencephalon. These data suggest that the ectopic vesicles induced by *EnR-Otx2* transfection seem to have the characteristics of the telencephalon.

## Discussion

### Repression of *Otx2* Function in Chick Eyes

As in the case of *Otx1^−/−^;Otx2^+/−^* mice [6], chick eyes displayed the formation of an “ectopic NR” in the outer layer of the OC, as a result of the severely impaired function of *Otx2*. The outer layer of the OC began to form an unpigmented-thick structure following *EnR-Otx2* transfection. In this tissue, the expression of some RPE-specific markers (Mitf, *Dct* and *MMP115*) was reduced but, instead, the expression of several differentiation markers of the NR (HuC/D and Islet1) was detected. In addition, the expression of *EnR-Otx2* also caused increases in cell proliferation and apoptosis in the developing eye, similar to *Otx1^−/−^; Otx2^+/−^* mice [6].

### Functions of *Otx2* are Associated with Regional Specification of the RPE

Both the RPE and NR are derived from the same developmental origin. As eye development proceeds, the presumptive RPE and NR regions become subdivided into the outer and inner layers of the OC, respectively. One focus of this study was to elucidate how Otx2 functions during these regional specifications of the RPE and NR.

We analyzed the expression patterns of factors contributing to NR development. Among them, Sox2 and *Fgf8* were ectopically expressed in the outer layer of the OC following *EnR-Otx2* transfection. In contrast, the expression of Pax6 seemed to be decreased in the ectopic NR, and there were no obvious changes in Six3 or Lhx2. Considering that Sox2 and FGF8 are known to induce NR cell fate in the outer layer of the OC (the presumptive RPE region) *in vivo* [11], [12], it is possible that Otx2 is required to repress the expression of these factors (Sox2 and *Fgf8*) in the outer layer of the OC (the presumptive RPE region). Correspondingly, in normally developing eyes, Sox2 and *Fgf8* are not detected in the outer layer of the OC (the presumptive RPE region) where Otx2 is expressed [11], [12].

**Figure 7:**
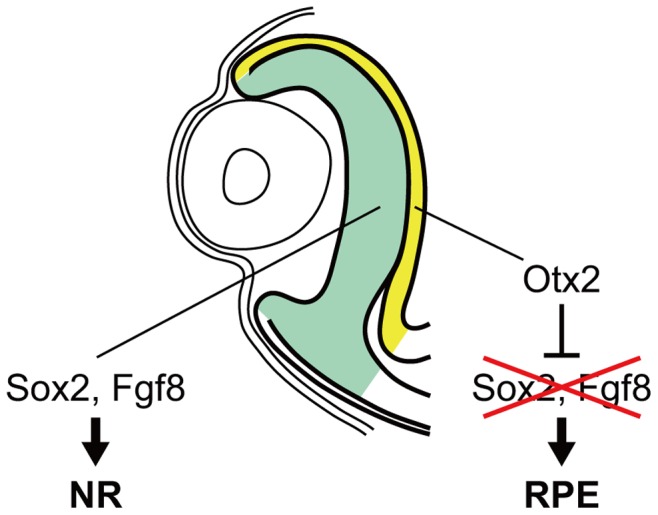
Schematic representation of how Otx2 functions in the regional specification of the RPE and NR in the OC. In developing chick eyes, Sox2 and *Fgf8* are expressed in the OC and induce NR differentiation. However, the expression domains of Sox2 and *Fgf8* are restricted to the inner layer of the OC, since Otx2 is expressed in the outer layer of the OC and represses the expression of Sox2 and *Fgf8*. As a result, the Sox2 and *Fgf8*-positive inner layer of the OC is induced to form the NR, whereas the Sox2 and *Fgf8*-negative outer layer is prevented from forming the NR and instead differentiates into the RPE.

Considering these data, we would like to propose the following hypothesis regarding the regional specification of the RPE and NR (Figure 7). In the normally developing OC, Sox2 and *Fgf8* function to induce NR differentiation. However, in the outer layer of the OC, the expression of Sox2 and *Fgf8* is repressed by Otx2, and Sox2 and *Fgf8* expression is restricted to and remains in the inner layer of the OC. As a result, NR differentiation is prevented in the outer layer of the OC and leads to formation of the RPE, whereas the NR is formed in the Sox2 and *Fgf8*-positive inner layer.

### Reduction of Pax6 Expression by *EnR-Otx2*

Our results show that the expression of Pax6 is reduced in the thickened outer layer/ectopic NR by *EnR-Otx2* transfection. However, Pax6 is known to induce ectopic NR formation in the RPE region [13], as do *Sox2* and FGF8. In the case of ectopic NR formation by FGF8, *Pax6* expression is initially absent, but emerges in the ectopic NR at a later stage of differentiation [11]. Therefore, we cannot exclude the possibility that Pax6 would also be expressed in the ectopic NR if the *EnR-Otx2*-transfected embryos were incubated for a much longer term. In fact, Martinez-Morales et al. reported that the ectopic NR of *Otx1^−/−^*; *Otx2^+/−^* mice is positive for Pax6 expression [6]. Moreover, it has been revealed that Pax6 promotes NR development [13], [42], [43], [44].

However, previous studies also indicate the requirement of Pax6 for RPE development, using chimeric mouse embryos composed of wild-type and *Pax6^Sey/Sey–Neu^*-mutant cells (both the *Sey* and *Sey^Neu^* alleles encode a non-functional Pax6 protein) [45]. In the outer layer of the OC of the chimera, the region occupied by Pax6-mutant cells shows an abnormally thickened-layer [45], suggesting that loss of function mutations in *Pax6* cause disruption of the mono-layered structure of the RPE. *Pax6* is also required to initiate Mitf expression in the developing eye, in a redundant manner with *Pax2* [46]. In addition, Pax6 is expressed in the presumptive RPE region (this study and [46]), and is also detected in cultured RPE cells which are derived from embryonic stem cells [47].

To elucidate the mechanisms of OC patterning in detail, it should be unveiled how Pax6 expression is regulated and how Pax6 switches its function according to the developmental context.

### Future Prospects

By incubating the embryos for a long term after *EnR-Otx2* transfection, ectopic vesicles were formed near the small eyes (Figure S2). Although the ectopic vesicles were continuously connected to the *EnR-Otx2* transfected small eyes, they lacked the characteristics of the RPE or NR. Instead, the expression of *Emx1*, Pax6 and *Nkx2.1* [39], [40], [41], [48], [49] suggests that the ectopic vesicles have the characteristics of the telencephalon. Although more detailed analyses on the molecular mechanisms involved are needed, our analyses of Otx2 function bring new insights into the relationships between eye and brain development.

EnR-Otx2 may bind to the Otx1 protein, since the structures of Otx1 and Otx2 are similar in their dimerization domain (homeodomain) and are able to bind the same DNA target sequences [50]. Moreover, the replacement of *Otx1* with *Otx2* rescues the phenotype of *Otx1* knock-out mice, at least in part [51]. Therefore, our observations reinforce the requirement for *Otx* genes in the development of chick eyes. Other techniques that selectively reduce *Otx2* or *Otx1* expression, such as RNA interference, would dissect the functional divergence of *Otx1* and *Otx2* in eye development.

In the developing eye, patterning the polarity of the OV along the dorsal-ventral and posterior-anterior axes is required for proper regional specification of the presumptive RPE and NR regions [52], [53], [54], [55]. In such a patterning process, BMP4 and Shh from the dorsal and ventral parts of the forebrain, respectively, are thought to be involved [56]. Moreover, Activin, BMP and Wnt from the extra-ocular mesenchyme or surface ectoderm are thought to regionalize the presumptive RPE [17], [18], [25], [26], as well as FGFs from the surface ectoderm to regionalize the presumptive NR [5], [21]. Future analyses should clarify how Otx2 mediates these signals from extra-ocular tissues to intrinsic molecular mechanisms in the OV and OC. By understanding how the expression domain of Otx2 is restricted to the presumptive RPE region, more details about the mechanisms responsible for regionalizing the RPE and NR in the developing eye will be unveiled.

## Materials and Methods

### Ethics Statement

All experiments involving animals were approved by the Nagahama Institute of Bio-Science and Technology (approval Id: 050).

### Chick Embryos

White Leghorn chicken eggs were incubated at 38°C. Developmental stages of embryos were assigned according to Hamburger and Hamilton [57].

### *In situ* Hybridization and Immunohistochemistry

*In situ* hybridization and immunohistochemistry were performed as previously described [3]. Primary antibodies used for immunohistochemistry include polyclonal antibodies against chicken Mitf (generated in our laboratory), Pax2 (COVANCE), phospho Histone-H3 (Upstate), Sox2 (MILLIPORE) and ssDNA (DAKO, Denmark), and monoclonal antibodies against HuC/D (Molecular Probes), Tuj1 (COVANCE), Islet1, neurofilaments, Pax6 and visinin (Developmental Studies Hybridoma Bank, DSHB, USA). Samples were observed using an Olympus BX51 microscope (Tokyo, Japan) with a cooled CCD camera.

### Electroporation

*In ovo* electroporation was carried out as described previously [3] with the following modifications. White Leghorn chicken eggs were incubated at 38°C until the chick embryos reached stage 9–11, according to Hamburger and Hamilton [57]. The plasmid solution was then injected into the OV. An anode (0.5 mm in diameter, 1.0 mm in length; Unique Medical Imada, Japan) and a cathode (tungsten needle) were placed on the outside and inside of the embryo, respectively, across the OV (and surface ectoderm). Rectangular pulses (7 V, 30 ms) were then charged twice using an electroporator (CUY, Tokiwa Science, Japan).

### Expression Vectors

The full-length chicken *Otx2* cDNA was inserted in the pMiwIII vector. In this pMiwIII-*wtOtx2* (*wtOtx2*) vector, *Otx2* is fused to a nucleic acid encoding FLAG-tag. pMiwIII-*Otx2-EnR* (*EnR-Otx2*) is constructed by modifying pMiwIII-*wtOtx2*, in which the chicken *Otx2* fragment with FLAG Tag is fused to a nucleic acid encoding the repressor domain of *Drosophila* Engrailed (corresponding to amino acids 1–298). Three to 4 µg/µl *Otx2-EnR* plasmids were injected into the OV for transfection.

### Transient Transfection Assays

*DCT* promoter activity, which is driven by *Otx2* (Takeda et al., 2003), was assessed by transient expression of reporter genes in D407 human RPE cells, as described previously (Takeda et al, 2003). Briefly, cells were cultured for 12–24 hr after plating in 12 well dishes, and then were transfected with pHDTL12 containing the luciferase gene under the control of the human *DCT* gene promoter, each expression plasmid and pRL-TK (an internal control) using the FuGENE 6 protocol (Roche Molecular Biochemicals). pRL-TK contains the herpes simplex virus thymidine kinase promoter region upstream of Renilla luciferase (Promega). Luciferase activity was measured using the Dual-LuciferaseTM Reporter Assay System (Promega). Reporter luciferase activity was normalized against Renilla luciferase activity.

## Supporting Information

Figure S1 **Increased cell proliferation and apoptosis in** ***EnR-Otx2*** **-transfected eyes.** Immunohistological analyses of cell proliferation (A-E) and apoptosis (F-J) in sections of normal eyes (A, D, F and I) and *EnR-Otx2*-transfected eyes (B, C, E, G, H and J). A and C-E indicate PHH3-positive mitotic cells (magenta), and C and E are merged images with GFP (green). F and H-J indicate ssDNA-positive apoptotic cells (magenta), and H and J are merged images with GFP (green). D is a highly magnified image of the box in A, as well as E of C, I of F, and J of H. B and G are bright field images of C and H, respectively. Open arrows and arrowheads in A and D indicate PHH3-positive cells in the RPE and NR of the normal eye, respectively. Arrowheads and arrows in E indicate PHH3-positive cells in ectopic NR and NR of *EnR-Otx2*- transfected eyes, respectively. Arrows in F and I indicate ssDNA-positive cells in the normal eye. Arrows in H indicate ssDNA-positive cells which are located in the *EnR-Otx2*-transfected areas. Arrowheads and arrows in J indicate ssDNA-positive cells in ectopic NR and NR of *EnR-Otx2*-transfected eyes, respectively. RPE, retinal pigment epithelium. NR, neural retina. Le, lens. Scale bars: 100 µm in A (for A-C and F-H); 50 µm in D (for D and I); 10 µm in E (for E and J).
(TIF)

Figure S2 **Ectopic formation of telencephalon-like vesicles following** ***EnR-Otx2*** **transfection.** (A) Lateral view of an embryo incubated for 1 week after *EnR-Otx2* transfection. The right eye displays a ‘small eye’ compared to the untransfected-left eye. The arrow indicates a vesicle which is ectopically formed adjacent to the small eye. (B-K) Immunohistological and *in situ* hybridization analyses of eye and brain markers. Sections in B-K are sliced along the plane indicated by the white line in A. Sections in B, D, H and K are stained with anti-Pax6 antibody (green) and DAPI (blue). Sections in C and E are stained with anti-Visinin antibody (green) and DAPI (blue). B, C, H and K indicate the tissues around the small eye and ectopic vesicles formed by *EnR-Otx2*. D and E indicate parts of the normally developing eye. Sections in F and I are stained with an antisense probe for *Emx1* (violet-blue). Sections in G and J are stained with an antisense probe for *Nkx2.1* (violet-blue). H and K are highly magnified images of boxes in B, as well as I of F, and J of G. F, G, I and J indicate tissues around the small eye (right eye), ectopic vesicle (Etc) and normally developing untransfected eye (left eye) of an *EnR-Otx2*-transfected embryo. Arrowheads in H and I indicate the dorsal area of ectopic vesicles in which both Pax6 and *Emx1* signals are detected. Arrowheads in J indicate the ventral areas of ectopic vesicles in which the *Nkx2.1* signal is detected. RPE, retinal pigment epithelium. NR, neural retina. Etc, Ectopic vesicle. inl, inner nuclear layer. onl, outer nuclear layer. gcl, glial cell layer.
(TIF)
