# Supplementary figures and images for: Otx2 Is Involved in the Regional Specification of the Developing Retinal Pigment Epithelium by Preventing the Expression of Sox2 and Fgf8, Factors That Induce Neural Retina Differentiation
Source: PLoS One. 2012 Nov 8;7(11):e48879. doi: 10.1371/journal.pone.0048879 (PMC3493611; doi:10.1371/journal.pone.0048879)

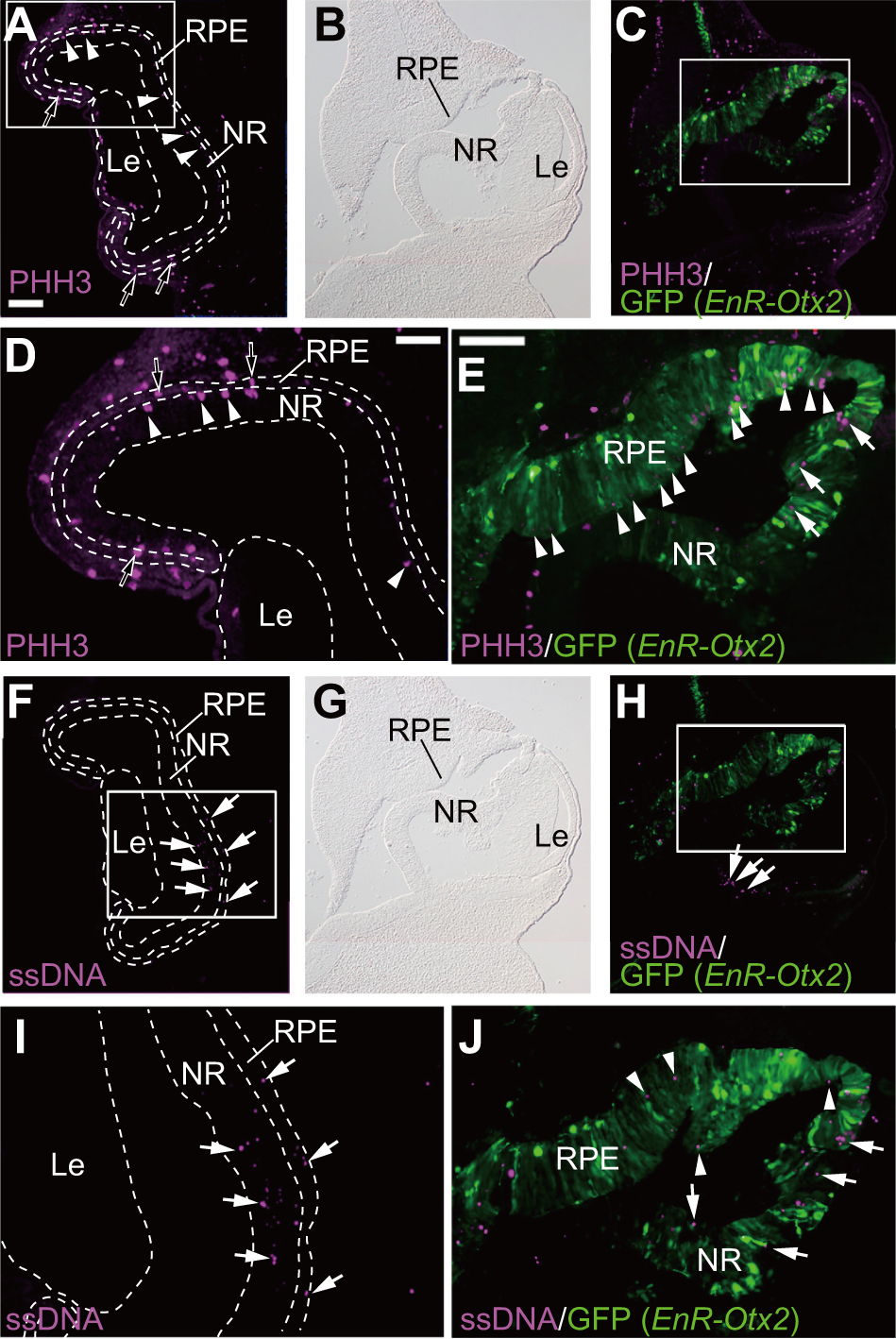

Supplement: Figure S1 — Increased cell proliferation and apoptosis in EnR-Otx2 -transfected eyes. Immunohistological analyses of cell proliferation (A-E) and apoptosis (F-J) in sections of normal eyes (A, D, F and I) and EnR-Otx2-transfected eyes (B, C, E, G, H and J). A and C-E indicate PHH3-positive mitotic cells (magenta), and C and E are merged images with GFP (green). F and H-J indicate ssDNA-positive apoptotic cells (magenta), and H and J are merged images with GFP (green). D is a highly magnified image of the box in A, as well as E of C, I of F, and J of H. B and G are bright field images of C and H, respectively. Open arrows and arrowheads in A and D indicate PHH3-positive cells in the RPE and NR of the normal eye, respectively. Arrowheads and arrows in E indicate PHH3-positive cells in ectopic NR and NR of EnR-Otx2- transfected eyes, respectively. Arrows in F and I indicate ssDNA-positive cells in the normal eye. Arrows in H indicate ssDNA-positive cells which are located in the EnR-Otx2-transfected areas. Arrowheads and arrows in J indicate ssDNA-positive cells in ectopic NR and NR of EnR-Otx2-transfected eyes, respectively. RPE, retinal pigment epithelium. NR, neural retina. Le, lens. Scale bars: 100 µm in A (for A-C and F-H); 50 µm in D (for D and I); 10 µm in E (for E and J). (TIF) [file pone.0048879.s001.tif]

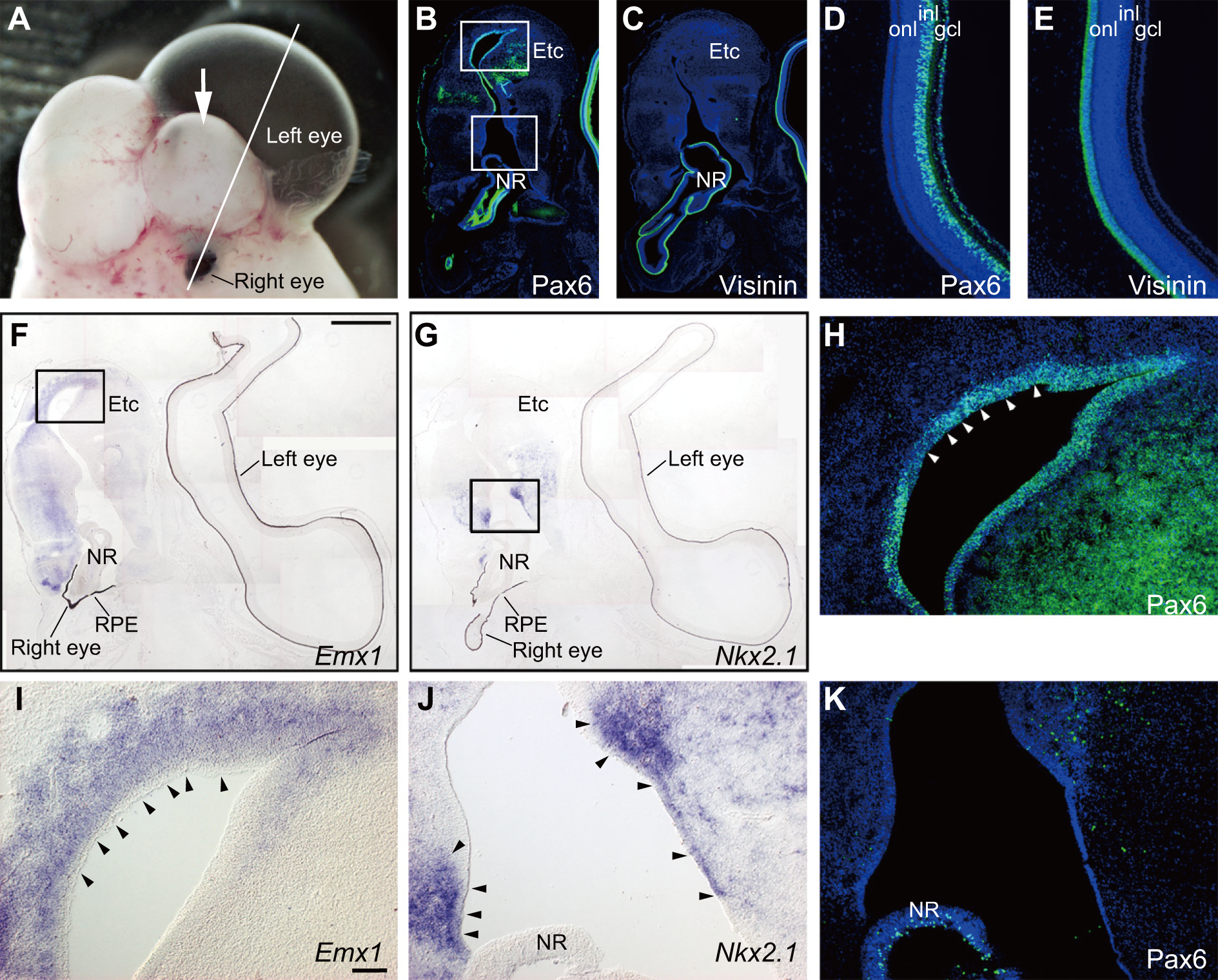

Supplement: Figure S2 — Ectopic formation of telencephalon-like vesicles following EnR-Otx2 transfection. (A) Lateral view of an embryo incubated for 1 week after EnR-Otx2 transfection. The right eye displays a ‘small eye’ compared to the untransfected-left eye. The arrow indicates a vesicle which is ectopically formed adjacent to the small eye. (B-K) Immunohistological and in situ hybridization analyses of eye and brain markers. Sections in B-K are sliced along the plane indicated by the white line in A. Sections in B, D, H and K are stained with anti-Pax6 antibody (green) and DAPI (blue). Sections in C and E are stained with anti-Visinin antibody (green) and DAPI (blue). B, C, H and K indicate the tissues around the small eye and ectopic vesicles formed by EnR-Otx2. D and E indicate parts of the normally developing eye. Sections in F and I are stained with an antisense probe for Emx1 (violet-blue). Sections in G and J are stained with an antisense probe for Nkx2.1 (violet-blue). H and K are highly magnified images of boxes in B, as well as I of F, and J of G. F, G, I and J indicate tissues around the small eye (right eye), ectopic vesicle (Etc) and normally developing untransfected eye (left eye) of an EnR-Otx2-transfected embryo. Arrowheads in H and I indicate the dorsal area of ectopic vesicles in which both Pax6 and Emx1 signals are detected. Arrowheads in J indicate the ventral areas of ectopic vesicles in which the Nkx2.1 signal is detected. RPE, retinal pigment epithelium. NR, neural retina. Etc, Ectopic vesicle. inl, inner nuclear layer. onl, outer nuclear layer. gcl, glial cell layer. (TIF) [file pone.0048879.s002.tif]
